# Areca Nut Chewing and an Impaired Estimated Glomerular Filtration Rate as Significant Risk Factors for Non-Muscle-Invasive Bladder Cancer Recurrence

**DOI:** 10.1038/srep29466

**Published:** 2016-07-07

**Authors:** Jian Cao, Ran Xu, Xiaokun Zhao, Zhaohui Zhong, Lei Zhang, Xuan Zhu, Shuiqing Wu, Kai Ai

**Affiliations:** 1 Department of Urology, The Second Xiangya Hospital, Central South University, 139 Middle Renmin Road, Changsha 410011, Hunan Province, People’s Republic of China; 2 MRC Centre for Reproductive Health, The Queen’s Medical Research Institute, 47 Little France Crescent, Edinburgh EH16 4TJ, United Kingdom

## Abstract

The present study sought to investigate the predictive value of preoperative clinicopathological variables, with a special focus on areca nut chewing, on disease recurrence and progression in patients with non-muscle-invasive bladder cancer (NMIBC). Data from 242 patients diagnosed with NMIBC between 2008 and 2013 were analyzed retrospectively. Fifteen clinicopathological variables were analyzed to evaluate their prognostic value. During a mean observation period of 21 months, disease recurrence occurred in 140 patients (57.9%). On multivariate analysis, heavy-areca nut chewing (HR = 2.18, 95% CI: 1.37–3.47), current smoking (HR = 3.09, 95% CI: 1.99–4.80), moderately impaired estimated glomerular filtration rate (eGFR) (HR = 1.76, 95% CI: 1.09–2.83), severely impaired eGFR (HR = 3.32, 95% CI: 1.70–6.48) and tumor grade (HR = 1.94, 95% CI: 1.36–2.77) were independent factors for recurrence, based on which a risk factor model was developed to stratify patients into high, medium and low risk groups. In conclusion, our study suggests that in addition to quitting smoking, quitting areca nut chewing may also reduce the risk of first recurrence in NMBIC patients, monitoring and preserving their renal function may be beneficial as well. Further prospective studies are needed to verify the prognostic significance of these factors and the risk stratification model in this population.

Globally, bladder cancer is the 11th most commonly diagnosed cancer and the 14th leading cause of cancer deaths^1^; in China, it is the most common urological cancer in both males and females^2^. Bladder cancer is a heterogeneous disease with varying oncological outcomes. Approximately 70–80% of newly diagnosed patients present with non-muscle-invasive bladder cancer (NMIBC); approximately 50–70% of these tumors recur, and 10–30% progress to muscle-invasive bladder cancer (MIBC), which is associated with a high risk of distant metastases^3^. Unfortunately, it remains a challenge to predict which patients will develop recurrence and progression to MIBC. Because of the repetitive recurrence, subsequent complications, and progression to MIBC, there is a need for lifetime surveillance and repeated treatments, which create a heavy economic burden on the healthcare system^4^ and a psychological burden on patients^5^. Recently, a more precise prediction of the recurrence risk of NMIBC has become a research focus. In previous studies, several pathological and clinical variables have been identified as risk factors for NMIBC recurrence^6^,^7^,^8^,^9^. Tumor multiplicity, prior recurrence rate, tumor grade, concomitant carcinoma *in situ* (CIS) and tumor size are considered the most important variables predicting recurrence^10^. Although these tumor-related intrinsic factors provide valuable prognostic information, it is also important to identify extrinsic risk factors for NMIBC recurrence.

Areca nut is used as a masticatory substance by approximately 600 million people worldwide, and it is particularly popular in south Asia and in the Pacific region^11^. Areca nut chewing has a history of more than 360 years in our province (Hunan Province, China)^12^. According to an epidemiological survey from a city in our province, 35% of the subjects chewed the areca nut in 1986^12^, and this habit is even more popular now, people typically chew the areca nut with additives, including bittern, cassia twig oil, and the kernel of areca nut; in addition, those who chew areca nuts also tend to smoke tobacco. Based on our long-term clinical observation, we suspected that areca nut chewing might be associated with an increased risk of NMIBC recurrence. However, no study to date has evaluated the relationship between areca nut chewing and NMIBC recurrence. Therefore, in the present study, we investigated the potential associations between fifteen clinicopathological variables and prognosis of NMIBC, the fifteen variables included renal function, smoking status, co-morbidities, etc. (which were studied in previous studies), with a special focus on eGFR and areca nut chewing, aiming at developing a risk group stratification model.

## Results

### Baseline characteristics of the subgroups

Among 2043 patients who were diagnosed with NMIBC in our hospital between 2008 and 2013, a total of 242 patients met the enrolment criteria. The mean follow-up was 21 months and ranged from 2 to 71 months. The mean patient age at diagnosis was 64.2 years and ranged from 35 to 81 years. The mean time to recurrence was 13.05 months and ranged from 2 to 34 months. Sixty-four patients (26.4%) were female. During the observation, the disease recurred in 140 patients (57.9%), and the tumors progressed in 19 of these patients (13.6%). Table 1 presents the clinicopathological characteristics of the 242 patients according to the outcome of recurrence. The percentage of smoking, areca nut chewing, impaired eGFR, elevated NLR and higher tumour grade were significantly higher in the recurrence group than in the non-recurrence group.

### Association between clinical parameters and recurrence

The univariate survival analysis revealed that light-areca nut chewing and heavy-areca nut chewing, former and current smoking, elevated NLR, medium-impaired eGFR, severe-impaired eGFR and tumor grade were associated with recurrence (p < 0.05, log-rank test; Table 2). However, elevated NLR, light-areca nut chewing, and former smoking failed to achieve significance in the multivariate survival analysis. Moreover, when we treated eGFR as continuous variable, eGFR was also a significant risk factor for recurrence (HR = 0.97, 95% CI: 0.97–0.98, p < 0.001 Table 2) which means with every 1 ml/min/1.73 m^2^ increased eGFR, the recurrence risk drops to 97% of its previous level. Age (<70 vs. ≥70 year), gender, diabetes, hypertension, elevated PLR, CRP level, tumor size, intravesical therapy agents and tumor multiplicity were not significant predictor in the univariate analysis (Table 2).

**Figure 1:**
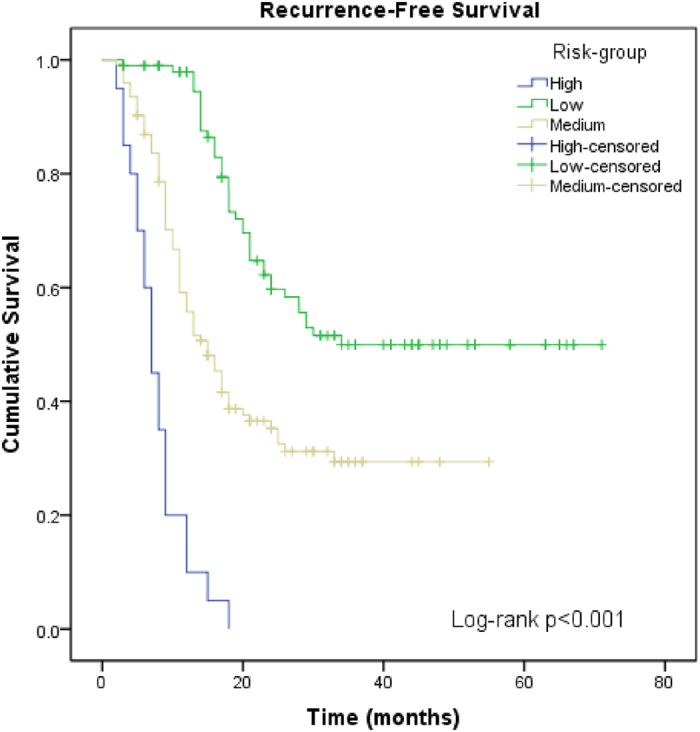
Kaplan-Meier analysis of the recurrence-free survival of NMIBC patients, stratified by risk group. Significance was determined for the comparisons of all group-pair combinations.

A risk calculation tool was developed to stratify the patients into high, medium, and low risk subgroups of recurrence, according to their eGFR, areca nut chewing history, tumor grade and current smoking. The regression coefficients for medium-eGFR, severe-eGFR, grade, heavy-areca nut chewing and current smoking were 0.562, 1.199, 0.663, 0.778 and 1.128 (Table 2), respectively. A point value for each significant risk factor was calculated by dividing each regression coefficient by the coefficient of the parameter with the highest regression coefficient, which was multiplied by the number of significant risk factors and rounded to the nearest integer. As a result, 99 patients (40.9%) scored zero, 123 patients (50.8%) scored 1, and 20 patients (8.3%) recorded scores of 2–3; these patients were then classified into the low-risk group, the medium-risk group and the high-risk group (Table 3), respectively. Figure 1 illustrates the Kaplan-Meier estimates for the subgroups according to the calculated risk of NMIBC recurrence. The mean recurrence-free survival time was 45.23 months (95% CI: 39.5–91) in the low-risk group, 27.7months (95% CI: 20.9–28.6) in the medium-risk group, and 7.8 months (95% CI: 6.0–9.5) in the high-risk group (Table 3).

### Association between the clinical parameters and progression

Tumor progression to a higher pT category or grade was observed in 19 patients, and 3 patients developed muscle-invasive disease. In the univariate analysis, only tumor grade (low vs. high) was a significant prognostic factor for tumor progression, with an HR of 3.26 (95% CI: 1.16–9.16). However, this factor did not reach significance in the multivariate analysis.

## Discussion

In this retrospective study involving 242 patients with non-muscle-invasive bladder cancer (NMIBC) who received transurethral resection (TUR), impaired eGFR, areca nut chewing, current smoking, and tumor grade were identified as independent risk factors for disease recurrence. Based on these findings, we developed a simple scoring system to stratify patients into low, medium and high risk groups for NMIBC recurrence.

Renal insufficiency was common in patients with NMIBC. Fifty-seven of the patients in our study (26.3%) had renal insufficiency, with an eGFR ≤60 ml/min/1.73 m^2^. However, this was only a single center report from a top academic hospital, which usually receives a greater proportion of cancer patients with complicated and severe diseases, possibly accounting for the substantially higher rate of patients with an impaired eGFR in our study. A previous study has reported a similar prevalence of impaired eGFR in cohorts of patients with NMIBC, correlating well with our study^13^.

Renal insufficiency, which is common in cancer patients^14^, has been considered to be a relevant clinical parameter for bladder cancer. On the one hand, both cancer and cancer treatments, including nephrotoxic chemotherapy agents, radiotherapy, and the contrast agents used in radiology, can lead to renal impairment. On the other hand, renal function can influence the choice of therapeutic strategies. Gupta N. *et al*.^15^ conducted a study in patients with bladder cancer and obstructive uremia and suggested that these patients may not require preoperative urinary diversion before radical cystectomy if their serum creatinine levels are less than 3 mg/d. The authors also found that the ileal conduit may not be ideal for patients with serum creatinine levels greater than 2.5 mg/dl, which would cause a deterioration of renal function^15^. In a retrospective study by Rausch S. *et al*.^13^, the authors have revealed that an impaired eGFR is a strong independent predictor of not only tumor recurrence but also tumor progression. In our study, we confirmed that eGFR is a significant predictive indicator of NMIBC recurrence. The reason why patients with renal insufficiency exhibit higher risk of bladder cancer recurrence may be because of an increased prevalence of immunosuppressive conditions in these patients. However, in our review of the literature, we found studies on immune dysfunction only in patients with end-stage renal disease (ESRD). Uremia is associated with a state of immune dysfunction characterized by immunosuppression, such as impaired T lymphocyte activation, an increased Th1/Th2 ratio, a decreased B lymphocyte count, a reduced ability of antigen-presenting cells to recognize tumor-associated antigens, hyporeactive monocytes, and decreased bactericidal abilities of neutrophils, indicating an unfavorable innate host defense mechanism^16^. The activation of hypoxia-induced factor (HIF) in chronic kidney disease might be another mechanism involved in cancer development. Hypoxia has been considered to be a final common pathway that leads to renal insufficiency, which is counteracted by defense mechanisms that prevent the activation of HIF, a transcription factor that is frequently activated in cancer^17^. Palit, V. *et al*.^18^ have reported a significant association of HIF-1α expression with recurrence and survival in NMIBC patients. Moreover, chronic bladder irritation and/or exposure to greater concentrations of some urinary carcinogenic substances resulting from chronic kidney disease or smaller amounts of urine for flushing out tumor cells from the bladder are also plausible reasons for recurrence in these patients^19^. Jørgensen *et al*. have observed that patients with a urine albumin to creatinine ratio of greater than 1.11 mg/mmol are 8.3-fold more likely to receive a diagnosis of bladder canceer^20^. Roth *et al*. have analyzed urine samples from patients with chronic kidney disease and healthy controls and have provided evidence for increased total CK-18 serum and urine levels in patients with chronic kidney disease, possibly indicating that epithelial cell necrosis is prevalent in chronic kidney disease^21^.

Diabetes and hypertension are the leading causes of chronic kidney disease in Chinese elderly patients, with glomerulonephritis as the major cause of ESRD in Chinese adults^22^, however, exact diagnosis is often difficult. The excess cancer rates were seen in glomerulonephritides^23^ and membranous nephropathy^24^. Diabetes was shown with a modestly increased risk of bladder cancer in a meta-analysis study^25^. However, in present study not all patients with renal impairment had a clear diagnosis for the cause, especially those with medium impaired eGFR. Moreover, considering the complexity in renal impairment etiology, it is unlikely that a study of current sample size have enough power to detect association between a specific renal impairment cause and the NMIBC recurrence.

To our knowledge, this is the first report of the correlation between areca nuts chewing and NMIBC recurrence. The multivariate survival analysis showed that the heavy-areca nuts chewing patients (HR = 2.18, 95% CI: 1.37–3.47) exhibited a significant increase in NMIBC recurrence. It is well established that the areca nut is a common human carcinogen^26^, particularly for oral cancer^27^. Areca nut products and derivatives, such as arecoline and areca nut-derived nitrosamines, also referred to as areca nut-specific nitrosamines, interact with DNA and other cellular targets and cause general carcinogenic effects^28^. The areca nut and/or its constituents (polyphenols and tannins) undergo metabolic activation and nitrosation, which together produce the ultimate carcinogenic derivatives. Then, these derivatives cause DNA mutations and strand breaks, as well as biochemical and structural changes that subsequently increase cell proliferation, pre-neoplastic changes and carcinogenesis^28^. Second, the expression patterns of tumor suppressor genes, such as TP53, BRCA1 and BRCA2, are strongly altered^28^ in people who overuse areca nuts; these alterations are likely to diminish their tumor suppressor properties and favor carcinogenesis. A study investigating the risk of p53 gene mutations in esophageal squamous cell carcinoma and the areca nuts chewing habit in Taiwanese patients has revealed that areca nut chewers exhibit a significantly higher incidence of p53 gene mutations than non-chewers^29^. In addition, areca nuts and their constituents can induce biochemical changes that promote cancer development. It has been shown that areca nut reduces the glutathione synthetase content in cells, leading to increased oxidative stress that can cause DNA damage and trigger several signaling pathways implicated in the carcinogenic process^28^. Areca nuts chewing also promotes tumor progression by inducing matrix metalloproteinase-2 and -9 secretion^28^. Moreover, areca nuts chewing induces a metabolic syndrome that might indirectly contribute to the development of bladder cancer. There is evidence suggesting that areca nuts chewing in conjunction with metabolic syndrome is harmful^30^. In turn, metabolic syndrome is a risk factor for urothelial carcinoma of the bladder^31^. Furthermore, the risk of carcinogenesis progressively increases in patients with continued areca nut exposure^28^. Notably, we noticed that this population usually has an unhealthy lifestyle, which might also be a contributor to tumor recurrence. These patients commonly chew the areca nut together with tobacco when staying up late playing cards or mah-jongg. However, most of this evidence has been derived from studies of oral and oropharyngeal cancer, and further laboratory experiments should be performed to investigate the its role in bladder cancer development.

Smoking is a strong, established risk factor for bladder cancer development, accounting for approximately 50% of cases^10^. In the present study, smoking status (never smoking vs. former smoking and current smoking) was associated with disease recurrence in the univariate analysis. However, after adjusting for those significant variables in the univariate analysis, including gender, areca nuts chewing, NLR, eGFR, tumour multiplicity, t-stage, and tumor grade, current smoking remained an adverse factor but former smoking did not. These results indicated that smoking cessation may reduce the risk of disease recurrence, which needs to be verified in a prospective study. Our results correlate well with those from previous studies. In a large prospective phase III study, Lammers R. *et al*.^32^ have revealed that smoking status, including ex-smokers and current smokers, is an independent significant factor for predicting the recurrence of NMIBC. Later, a subsequent study by Rink M *et al*. has provided evidence that smoking cessation reduces the risk of disease recurrence. The authors have found a significant dose-response relationship between cumulative smoking exposure when combining smoking quantity and duration and the clinical outcomes of NMIBC patients^8^. Our study further confirmed the detrimental effects of cigarette smoking on oncological outcomes of patients with NMIBC.

In previous studies, several inflammatory indices obtained from blood tests, including C-RP^33^, NLR^7^, and albumin levels^34^, have been found to be associated with the treatment outcomes of patients with bladder cancer. Among those markers, NLR has been identified as an independent predictor of disease recurrence in patients with NMIBC, with 3-year recurrence-free survival rates of 27% and 56% for patients with NLR >2.43 and ≤2.43, respectively^7^. However, Demirtas *et al*.^35^ have revealed no significant association between elevated NLR (≥2.5) and overall survival in patients who had undergone radical cystectomy. In our study, we found that patients with an NLR ≥2.5 (HR = 1.67, 95% CI: 1.20–2.33) had an approximately 1.7-fold greater risk of recurrence compared with those with an NLR <2.5 in the univariate analysis. However, there was no significant association between the groups in the multivariate analysis. The discrepancies between these inconsistence results probably arose from the heterogeneity of the patients, different operations and intravesical therapy agents, and the sizes of the cohorts, as well as the optimal NLR cut-off value. Some studies have shown that C-RP is a prognostic factor in metastatic bladder cancer and MIBC^33^,^36^. Hendrik Eggers *et al*. have evaluated the prognostic role of the pretreatment serum C-RP levels in a retrospective study of 34 patients. The authors have revealed that a C-RP level ≥80 mg/l is an independent risk factor for poor overall survival^33^. Yoshida *et al*.^36^ have defined an elevated C-RP level as >0.5 mg/dl and have found that patients with a C-RP level above the cut-off have a significantly shorter cancer-specific survival than the remaining patients. However, we did not find a significant association between elevated C-RP levels and NMIBC recurrence. This result might be because the NMIBC is mainly limited to the mucosal layer of the bladder and thus has a minimal influence on the systemic inflammatory status^13^. Although PLR has been described to be associated with a poor prognosis in a variety of malignancies, no such study has performed in NMIBC. Therefore, we also evaluated the prognostic role of the pretreatment PLR level in patients with NMIBC. No significant association between elevated PLR levels and the recurrence of NMIBC was identified. A more optimal prognostic PLR cut-off value should be identified, and larger prospective cohorts are also required to evaluate the prognostic value of PLR in NMIBC patients. Although diabetes and hypertension failed to achieve significant predictor status in the univariate and multivariate analyses, these two variables were significantly associated with impaired eGFR and areca nut chewing. The interrelations between these conditions might potentially lead to increased recurrence and progression through renal insufficiency and areca nut chewing. Of note, our study did not identify tumor multiplicity and tumor size as risk factors of recurrence, which have been found to be significantly predictive in the European Organization of Research and Treatment of Cancer NMIBC risk tables^10^.

Our study has several limitations inherent to its small sample size and retrospective design. Areca nut chewing and smoking history were self-reported and therefore were subject to recall bias. Future studies using biochemical verification of smoking and areca nut chewing status are needed to determine the extent of misreporting among NMIBC patients. Additionally, a synergetic effect between areca nut chewing and tobacco use in causing solid cancers were identified^37^; unfortunately, the small sample size in our study prohibited us from conducting such an analysis. Finally, due to the short follow-up time and the low mortality rate of NMIBC in general we could not analyze patients’ survival.

In conclusion, our study delivers important information to both NMIBC patients and their health care providers, on predicting the prognosis of NMIBC and postoperative management of the disease. With the evidence gained from our study, we strongly recommend our patients to quit areca nut chewing and smoking. Since impaired renal function is a risk factor for NMIBC recurrence, we need to watch more closely on those patients with renal insufficiency. However, to what extent quitting areca nut chewing and smoking, as well as preserving renal function can reduce NMIBC reoccurrence remain to be further studied prospectively, we are planning a cohort study which compares the recurrence rates and cost-effectiveness between patients who are given intervention on the risk factors and those who receive conventional treatment.

## Patients and Methods

### Study population

A retrospective review of medical records was performed to identify patients with NMIBC who had received transurethral resection (TUR) at the Second Xiangya Hospital, Central South University between 2008 and 2013. The study was approved by the Institutional Review Board and was performed in accordance with the Declaration of Helsinki. The inclusion criteria for patients to be eligible for the study were as follows: a: histological diagnosis of pure urothelial carcinoma without a history of other malignancies; b: use of TUR as the initial treatment; c: pathologic stage Ta or T1 (all T1 specimens included the muscle layer); and d: complete and valid clinical and follow-up data available. Patients who had undergone chemotherapy after TUR and those who had been diagnosed as CIS were excluded. According to published literatures, dialysis and renal transplantation themselves are independent risk factors for cancers, including bladder cancer^38^,^39^,^40^. In addition, the eGFR values in these patients cannot reflect their real renal function. Therefore, in order to reduce the interference of these two risk factors on a true relationship between eGFR and NMIBC recurrence, we excluded patients with dialysis and renal transplantation. On the basis of the criteria described above, a total of 242 subjects were identified from 2043 NMIBC patients who were diagnosed between 2008 and 2013 in our hospital.

### Data collection and definitions

Fifteen patient demographic and clinicopathological features were reviewed (Table 1). Staging and grading were performed according to the Union for International Cancer Control TNM system and the International Society of Urologic Pathology (2004 version) classification system, respectively. Renal function was measured using eGFR, which was calculated using the equation for estimating the modified glomerular filtration rate for Chinese patients [eGFR (ml/min/1.73 m^2^) = 175 × Scr − 1.234 × age − 0.179 (×0.79 if female)]^41^; an eGFR <60 ml/min/1.73 m^2^ was used as the threshold value for impaired renal function. Patients with an impaired preoperative eGFR who had recovered to an eGFR ≥60 ml/min/1.73 m^2^ due to concomitant hydronephrosis at TUR and normalization of renal function after surgery or stent placement were excluded from the impaired eGFR group. An elevated CRP level was defined as a serum level greater than 0.5 mg/dl, according to a previous study^36^. In the present analysis, the cut-off values for NLR and PLR were 2.5 and 190, respectively, according to their median values, in all patients. On the basis of their areca nut chewing history, the patients were divided into three groups: non-areca nut chewing; light-areca nut chewing, fewer than 10 nuts per day for at least 1 year before TUR; and heavy-areca nut chewing, more than 10 nuts per day for at least 1 year before TUR. The patients were divided into three groups according to their preoperative eGFR values: normal, eGFR ≥60 ml/min/1.73 m^2^, moderately-impaired eGFR, eGFR 60–20 ml/min/1.73 m^2^ and severely-impaired, eGFR <20 ml/min/1.73 m^2^. Patients with different smoking histories were divided into three groups: non-smokers; former-smokers: quit at least 1 year before TUR; and current-smokers: at least one year of smoking history before TUR. Acute urinary tract infection was ruled out preoperatively by a urine analysis in all patients.

The drugs we used for intravesical therapy were mitomycin, epirubicin and gemcitabine. We did not use BCG because it was only approved by the China Food and Drug Administration very recently and has not been regularly used in our hospital yet. The treatment protocol consisted of immediate postoperative instillation, followed by an 8-week induction course, with a maintenance schedule once every three months for up to 1–2 years for all patients. Patient’s follow-up was performed in an outpatient setting, according to the recommendations of the respective contemporary guidelines (European Association of Urology Guidelines). The follow-up protocol was designed based on the risk level of NMIBC (Supplemental Table 1). Recurrence-free survival was defined as the period between TUR and the detection of the first local recurrence or distant metastasis or the end of the study. Recurrence was defined as tumor recurrence after TUR, with or without pathological upstaging or upgrading. When a patient showed pathological progression by either upstaging or upgrading, tumor progression was recorded. Patients who died of any causes prior to recurrence, who had not developed recurrence at the end of 2013 or who were lost to follow-up were treated as censored cases.

### Statistical analyses

The data were analyzed using SPSS version 19.0 (SPSS Inc., Chicago, IL, USA). The associations of the clinicopathological covariates between different groups were evaluated with the chi-squared test. A univariate Cox proportional hazard regression analysis was performed to assess the individual risk factors. Those factors with an association with the dependent variable at p < 0.05 were included in a multivariate Cox regression model. An outcome prediction model was developed on the basis of the regression coefficients from the final multivariate model with the “enter” method. The recurrence-free survival rates of patients stratified by risk group were determined by the Kaplan-Meier method, and the difference was determined with the log-rank test. In all cases, a two-tailed p < 0.05 was considered significant.

### Ethics approval and consent to participate

The study was approved by the Institutional Review Board and was performed in accordance with the Declaration of Helsinki.

## Additional Information

**How to cite this article**: Cao, J. *et al*. Areca Nut Chewing and an Impaired Estimated Glomerular Filtration Rate as Significant Risk Factors for Non-Muscle-Invasive Bladder Cancer Recurrence. *Sci. Rep*. **6**, 29466; doi: 10.1038/srep29466 (2016).

## Supplementary Material

Supplementary Information

## Figures and Tables

**Table 1:** Characteristics of 242 patients with NMIBC.

Variables	Non-recurrencen (%)	Recurrencen (%)	*P*
**Total**	102(42.1)	140(57.9)	
**Age (years)**			0 .103
<70	72(29.8)	84(34.7)	
≥70	30(12.4)	56(23.1)	
**Sex**			0 .437
Female	28(11.6)	36(14.9)	
Male	74(30.6)	104(43.0)	
**Diabetes**			0.519
No	72(29.8)	98(40.5)	
Yes	30(12.4)	42(17.4)	
**Hypertension**			0.335
No	72(29.8)	94(38.8)	
Yes	30(12.4)	46(19.0)	
**Areca nut chewing**			**<0.001**
Non	77(31.8)	68(28.1)	
Light	19(7.9)	29(12.0)	
Heavy	6(2.5)	43(17.8)	
**Smoking history**			**<0 .001**
Non	74(30.6)	62(25.6)	
Former	21(8.7)	41(16.9)	
Current	7(2.9)	37(15.3)	
**C-RP**			0.435
<0.5 mg/dl	48(19.8)	74(30.6)	
≥0.5 mg/dl	54(22.3)	66(27.3)	
**NLR**			**0 .002**
<2.5	72(29.8)	71(29.3)	
≥2.5	30(12.4)	69(28.5)	
**PLR**			0.896
<190	58(24.0)	78(32.2)	
≥190	44(18.2)	62(25.6)	
**eGFR**			**<0.001**
≥60 ml/min	92(38.0)	94(38.8)	
60–20 ml/min	8(3.3)	35(14.5)	
<20 ml/min	2(0.8)	11(4.5)	
**Tumor multiplicity**			0.436
1–2	53(21.9)	80(33.1)	
≥3	49(20.2)	60(24.8)	
**Tumor size**			0.363
<3	58(24.0)	71(29.3)	
≥3 cm	44(18.2)	69(28.3)	
**T-stage**			0.432
Ta	55(22.7)	83(34.3)	
T1	47(19.4)	57(23.6)	
**Grade**			**0.024**
low	71(29.3)	77(31.8)	
high	31(12.8)	63(26.0)	
**Intravesical agents**			0.305
Mitomycin	25(10.3)	31(12.8)	
Epirubicin	48(19.8)	56(21.9)	
Gemcitabine	29(12.0)	53(21.9)	
**Follow-up time(months)**			
Mean(rang)	31.3(3–71)	13.1(2–34)	

Percentage of the total 242 patients; **C-RP**: C-reactive protein.

**NLR**: neutrophil to lymphocyte ratio; **PLR**: platelet to lymphocyte ratio.

**eGFR:** estimated glomerular filtration rate.

**Table 2:** Results from the univariate and multivariate analyses of recurrence.

	Univariate HR(95% CI)	*p*	Multivariate HR(95% CI)	*p*	Regression
					Coefficient
**Age (years)**(<70 vs. ≥70)	1.27(0.91–1.79)	0.163			
**Sex** (Female vs. Male)	1.22(0.83–1.78)	0.314			
**DM** (Yes vs. No)	1.08(0.75–1.56)	0.665			
**Hypertension** (Yes vs. No)	1.06(0.75–1.51)	0.735			
**Areca nut chewing habits**					
Non (reference)					
Light	**1.69(1.09–2.61)**	**0.019**			
Heavy	**3.05(2.07–4.50)**	**<0.001**	**2.18(1.37–3.47)**	**0.001**	**0.778**
**Smoking habits**					
Non (reference)					
Former	**1.70(1.15–2.52)**	**0.008**			
Current	**4.10(2.71–6.20)**	**<0.001**	**3.09(1.99–4.80)**	**<0.001**	**1.128**
**C-RP** (<0.5 vs. ≥0.5 mg/dl)	1.03(0.74–1.44)	0.852			
**NLR** (<2.5 vs. ≥2.5)	**1.67(1.20–2.33)**	**0.003**			
**PLR** (<190 vs. ≥190)	1.10(0.79–1.54)	0.573			
**eGFR**					
>60 ml/min (reference)					
60–20 ml/min	**2.77(1.86–4.12)**	**<0.001**	**1.76(1.09–2.83)**	**0.021**	**0.562**
<20 ml/min	**4.19(2.22–7.89)**	**<0.001**	**3.32(1.70–6.48)**	**<0.001**	**1.199**
**eGFR (continuous)**					
per 1 mL/min/1.73 m^2^	**0.97(0.97–0.98)**	**<0.001**			
**Tumor multiplicity** (1–2 vs.≥3)	1.20(0.86–1.68)	0.282			
**Tumor size** (<3 cm vs. ≥3 cm)	1.15(0.40–1.61)	0.398			
**T-stage** (Ta vs. T1)	1.23(0.88–1.73)	0.226			
**Grade** (low vs. high)	**1.93(1.38–2.70)**	**<0.001**	**1.94(1.36–2.77)**	**<0.001**	**0.663**
**Intravesical agents**					
Mitomycin (reference)					
Epirubicin	0.77(0.50–1.20)	0.254			
Gemcitabine	0.78(0.50–1.21)	0.262			

P values in bold indicate a significant difference. Regression coefficient from Multivariate Cox analysis. **HR**: hazard ratio.

**C-RP**: C-reactive protein; **NLR**: neutrophil to lymphocyte ratio; **PLR**: platelet to lymphocyte ratio; **eGFR**: estimated glomerular filtration rate.

**Table 3:** Risk group classifications.

Risk Group	Prognostic Score	Patients n (%)	Mean time to recurrence (months)
Low	0	99(40.9)	45.23(95% CI: 39.54–50.91)
Medium	1	123(50.8)	24.74(95% CI: 20.9–28.59)
High	2–3	20(8.3)	7.75(95% CI: 5.97–9.53)

The prognostic score was calculated as follows: eGFR (60–20 ml/min) × 0.56/1.199 + eGFR (<20 ml/min) × 1.199 /1.199 + Current-Smoker × 1.128/1.199 + Heavy-areca nut chewing × 0.778/1.199 + Grade-high × 0.663/1.199.
