# Supplementary material for: Areca Nut Chewing and an Impaired Estimated Glomerular Filtration Rate as Significant Risk Factors for Non-Muscle-Invasive Bladder Cancer Recurrence
Source: Sci Rep. 2016 Jul 7;6:29466. doi: 10.1038/srep29466 (PMC4935873; doi:10.1038/srep29466)
Supplement: Supplementary Information [file srep29466-s1.pdf]

Areca Nut Chewing and an Impaired Estimated Glomerular Filtration Rate as Significant Risk Factors for Non-Muscle-Invasive Bladder Cancer Recurrence

Jian Cao[1 2], MD; Ran Xu[1 *], PhD; Xiaokun Zhao[1],PhD; Zhaohui Zhong[1],PhD; Lei Zhang[1],PhD; Xuan Zhu[1], PhD; Shuiqing Wu[1],MD; and Kai Ai[1], MD

[1] Department of Urology, The Second Xiangya Hospital, Central South University, 186 Middle Renmin Road, Changsha 410011, Hunan Province, People's Republic of China; [2]MRC Centre for Reproductive Health, The Queen's Medical Research Institute, 47 Little France Crescent, Edinburgh EH16 4TJ, United Kingdom.

[*]Corresponding author: Ran Xu (Email: xuran@csu.edu.cn; Telephone: 0086 186-0841-8000; Fax Number: 0086 0731-85533525)

Supplemental table 1-Follow-up protocol of NMIBC for patients after TUR

	Low-grade	High-grade
Cystoscopy	3 months after TUR. If negative, subsequent cystoscopy is advised 9 month later, and then yearly for 5 year	3 months after TUR. If negative, subsequent cystoscopy and cytology should be repeated every 3 months for a period of 2 year, and every 6 months thereafter until 5 year, and then yearly
Urinary Cytology	Not recommended	3 months after TUR. If negative, subsequent cystoscopy and cytology should be repeated every 3 months for a period of 2 year, and every 6 months thereafter until 5 year, and then yearly
CT-IVU or IVU	Not recommended	yearly
Regular Laboratory Test/ Chest X-ray	With cystoscopy	With cystoscopy
